# Extremely low-frequency electric field suppresses not only induced stress response but also stress-related tissue damage in mice

**DOI:** 10.1038/s41598-020-76106-1

**Published:** 2020-12-07

**Authors:** Shinji Harakawa, Takaki Nedachi, Hiroshi Suzuki

**Affiliations:** 1 Bio-Self-Regulating Science Laboratory, Obihiro University of Agriculture and Veterinary Medicine, Inada, Obihiro, 080-8555 Japan; 2 Hakuju Institute for Health Science, Tokyo, Japan

**Keywords:** Therapeutics, Preventive medicine, Biomedical engineering, Neuroscience, Physiology, Endocrinology

## Abstract

Although extremely low-frequency electric fields (ELF-EF) have been utilised for therapeutic purposes, the biological effect and the underlying mechanism of ELF-EF have not been elucidated. Here, we developed a mouse model of immobilisation-induced increase in glucocorticoid (GC) to evaluate the effect of ELF-EF. Mice were exposed to 50-Hz 10 kV/m EF via a parallel plate electrode and immobilised as needed. The ELF-EF suppressed the immobilisation-induced increase in blood GC level. Here, the results of 32 tests using the model were pooled and analysed. The suppressive effect of ELF-EF on immobilisation-induced increase in GC was reproduced, and the GC level was slightly higher in the ELF-EF-treated mice than in the sham-controlled mice, a novel observation. The immobilisation-induced increase in lactate dehydrogenase, glutamic oxaloacetic transaminase, and glutamic pyruvic transaminase, markers of tissue damage, was suppressed by co-treatment with EF in the biochemical tests using the same plasma sample. In the metabolome analysis, the changes in corticosterones, leukotrienes, and hydroxyeicosatetraenoic acids, markers of inflammation, showed a pattern similar to that of the plasma GC level. Thus, ELF-EF suppresses the stress response that causes an increase in the GC level and slightly promotes GC production in the absence of stress. Moreover, the suppressive effect of ELF-EF on induced stress response might be involved in stress-induced tissue damage or inflammation in immobilised mice. Overall, the model and the data help explore the biological effect of ELF-EF and explain the stress-relieving effect of EF. They would be useful in determining the medical applications of EF in humans and animals.

## Introduction

The number of non-ionising electromagnetic fields (EMFs) has rapidly increased worldwide, and they have been verified as potential health risks by the International Commission on Non-Ionizing Radiation Protection (ICNIRP) and the World Health Organization^1–3^. Available data do not indicate whether the effects of low-frequency electric fields (EF) and/or magnetic fields on the neuroendocrine system have an adverse effect on human health^2^. The medical applicability of EMFs have been explored, and the potential of non-ionising EMFs for medical purposes has garnered interest recently. Although several established and familiar treatment methods and devices involving EMF have been used globally, our understanding of the biological effects of EMF exposure in medical applications is insufficient, such as transcutaneous electric nerve stimulation for neurological applications; vagal nerve stimulation; and transcranial magnetic stimulation for the treatment of depression, epilepsy, dementia, or pain^2,4^.

The following are some reasons for increasing ELF-EF research. First, the potential clinical effect of ELF-EF on unspecified complaints such as pain and insomnia of unknown origin, which are not covered by major medical care^5^. Second, it involves a non-pharmacological strategy (unlike drugs) that does not involve the administration of foreign substances into the body^6^. Finally, the methods are simple (the processes are hardly invasive)^5,6^. Non-pharmacological strategies are common in physiotherapy, but ELF-EF treatment differs from other methods in its ease of application. Compared with other electrophysiological treatments such as attaching electrodes to the affected area or wearing headgear, ELF-EF treatment can be performed by placing the body between two electrodes. The convenience of the method provides patients or physicians an option that does not compete with existing medical treatments, that is, it does not deprive existing therapeutic opportunities. Therefore, improving our understanding of mechanisms underlying the biological effects of ELF-EF will enable efficient treatment with ELF-EF, by identifying individuals who can benefit from ELF-EF therapy and adjusting the specification of EF in treatment methods. In addition, such an effort may provide a basis for reconsidering the low-safety concerns of ELF-EF.

Previously, we reported that ELF-EF can modulate energy metabolism^7–10^ and cell signalling pathways^11^ of the endocrine^7,12^ and immune systems^13^, and control behaviour^14,15^. Although there is a research gap in ELF-EF treatment as a protective modality for childhood leukaemia according to the ICNIRP guidelines^2^, the focus of our present study was to elucidate the effect of ELF-EF on stress response because of its reproducibility in the last decade.

Corticosterone is the main glucocorticoid (GC) produced by the adrenal gland. It regulates the expression of the corticotropin-releasing factor and proopiomelanocortin genes^16^; an increase in the corticosterone level generally reflects the physiological state of stress^17^.

In our previous study, using parallel electrodes to generate an EF, it was found that in BALB/c mice, the immobilisation-induced increase in GC level was reduced by exposure to 50 Hz EF when the voltage was applied via the upper electrode of a parallel plate electrode system. This effect was dependent on both intensity (kV/m) and exposure time^18^. Furthermore, EF did not shift the peak of the time-dependent increase in plasma GC level in immobilised mice but just reduced it^18,19^, and this effect depended on the configuration of the EF exposure system. For example, the immobilisation-induced increase in the GC level was significantly decreased in mice exposed to 1 kV/100 mm EF for 60 min (*P* < 0.01), but not in mice exposed to 0.5 kV/50 mm or 2 kV/200 mm EF, although these systems had the same EF strength^20^. Subsequently, the suppressive effect of EF exposure depended on the body surface area of mice exposed to the EF^21^, and the effect occurred regardless of sex or age^22^. Finally, the study model met the requirement to elucidate the biological effects of ELF including power line-frequency EF for quantitative and qualitative assessments, and the results revealed that the ELF-EF suppresses the induced stress response. There were no significant changes in the GC level in the absence of stress although a weak change trend was observed in our previous tests.

With the progress in understanding the biological effects of ELF-EF, new issues have emerged. (i) Although survey results have showed the clinical effect of electric field on indefinite complaints, there is no direct evidence related to the suppressive effect on stress response^18,22^. (ii) As EMF is a biohazard, it is suspected that fields act as stressors or cause damage to tissues, cells, or DNA^1,2^. The phenomena of (i) and (ii) are completely opposite. (iii) Although the stress response is suppressed by ELF-EF and the phenomenon can be changed according to the ELF-EF specifications and biological factors, there is no information about the input route or cause, why the phenomenon occurs, and the mechanism underlying the phenomenon. Furthermore, it has been hypothesised that surface stimulation and induced currents are triggers^1,2^, but their association with stress responses is not clear.

Most experiments performed using the model in our previous studies comprised the following four common groups: control, stress(−)/EF(−); EF alone, stress(−)/EF(+); immobilisation alone, stress(+)/EF(−); and co-treatment, stress(+)/EF(+). There are 32 experiments based on this model, including 19 that shared the four common groups and those with unpublished results. Furthermore, the four treatments were performed daily during the experimental period, and all experimental data could be analysed as one large data set. Here, the results of the tests performed using the model, including previously published results, were pooled and analysed, and some supplemental measurements were added to elucidate the potential medical application of ELF-EF.

## Results

### Effect of EF on the plasma GC level

**Figure 1:**
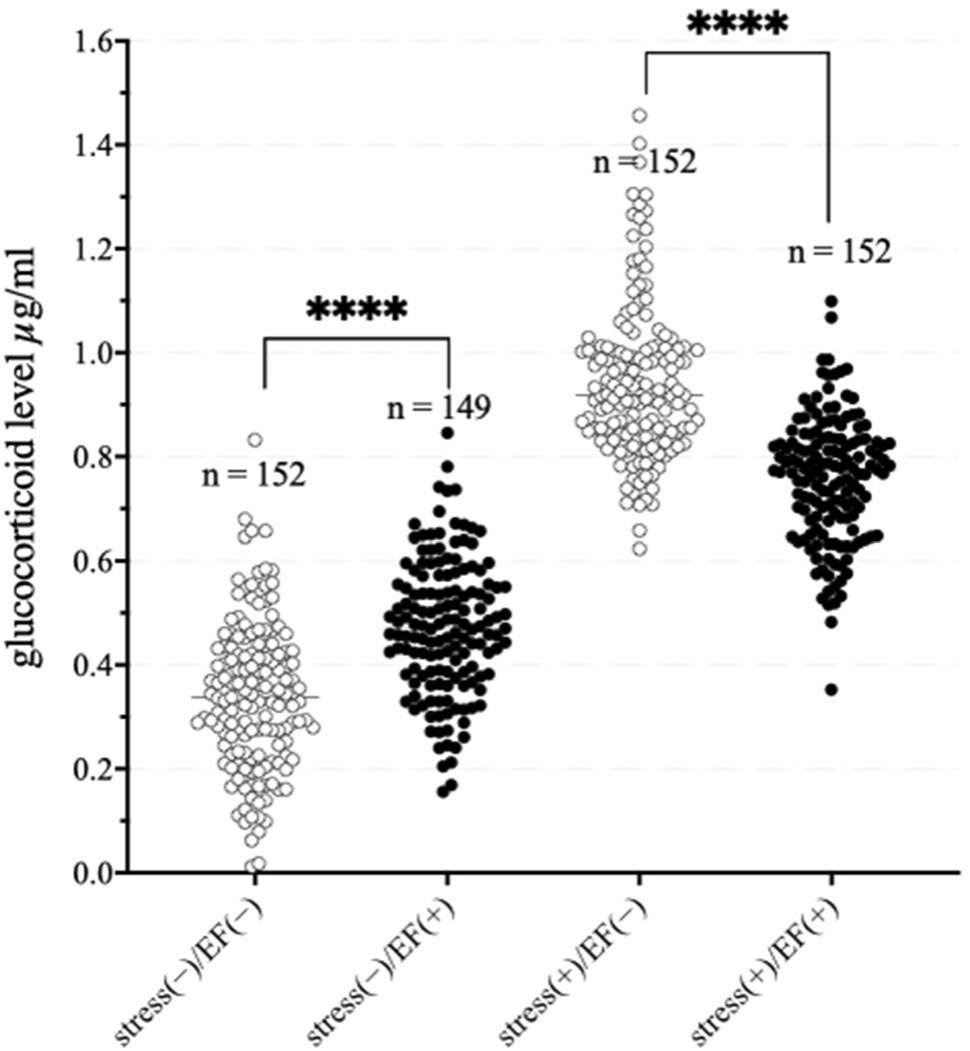
Effect of immobilisation and electric field exposure on immobilisation-induced increase in the plasma glucocorticoid level. Data of 19 tests conducted from 2015 to 2019 (number of tests: 14–32, n = 149–152) were pooled.

The GC data from 19 tests conducted between 2015 and 2019 (Table 1, note a), in four common groups: control, stress(−)/EF(−); EF alone, stress(−)/EF(+); immobilisation alone, stress(+)/EF(−); and co-treatment, stress(+)/EF(+) (n = 152, 149, 152, and 152, respectively), were pooled and analysed using a normality test (Kolmogorov–Smirnov test), Kruskal–Wallis test, and Dunn's multiple comparison test. The results of Kolmogorov–Smirnov test indicated that the data were non-normally distributed. The results of Kruskal–Wallis test were significant (*P* < 0.0001; Fig. 1). The results of Dunn's multiple comparison test revealed a significantly higher GC level in the stress(−)/EF(+) group than in the stress(−)/EF(−) group (*P* < 0.0005; Fig. 1). The GC level in the stress(+)/EF(+) group was also significantly higher than that in the stress(−)/EF(−) group (*P* < 0.0001), and its level in the stress(+)/EF(+) group was lower than that in the stress(+)/EF(−) group (*P* < 0.0001; Fig. 1).

**Table 1:** Date and summary of information of each pooled experiment conducted from 2014 (2011) to 2019.

Test no.	Date of GC assay	stress(−)/EF(−)			stress(−)/EF(+)			stress(+)/EF(−)			stress(+)/EF(+)			Note	References
		Mean	SD	N	Mean	SD	N	Mean	SD	N	Mean	SD	N		
1	18-Sep-2011	0.248	0.108	8				0.808	0.093	8	0.500	0.160	8	b	u.p. *
2	13-Jan-2012	0.147	0.106	8				0.923	0.068	10	0.665	0.122	8	b	^18^
3	8-Mar-2012	0.164	0.134	6				0.626	0.099	6	0.470	0.062	6	b	u.p.
4	22-May-2012	0.135	0.116	6				0.719	0.076	6	0.563	0.076	6	b	^20^
5	19-Jun-2012	0.088	0.013	5				0.623	0.067	5	0.432	0.094	5	b	u.p.
6	1-Oct-2012	0.178	0.062	6				0.883	0.155	5	0.588	0.120	6	b	^20^
7	16-Jan-2013	0.129	0.065	8				0.811	0.074	8	0.595	0.101	8	b	u.p.
8	27-May-2013	0.203	0.067	8				0.731	0.150	8	0.598	0.132	8	b	u.p.
9	23-Jul-2013	0.368	0.132	8				0.819	0.119	8	0.562	0.121	8	b	u.p.
10	13-Dec-2013	0.239	0.100	8				0.815	0.208	8	0.386	0.152	8	b	^20^
11	20-Mar-2014	0.269	0.169	8				0.969	0.177	8	0.540	0.266	8	b	^18^
12	4-Jun-2014	0.385	0.126	8				0.904	0.141	8	0.613	0.191	8	b	^20^
13	9-Oct-2014	0.255	0.160	8				0.924	0.283	8	0.592	0.166	8	b	u.p.
14	3-Jun-2015	0.519	0.192	8	0.675	0.089	6	1.157	0.207	8	0.789	0.119	8	a, b	^22^
15	1-Apr-2015	0.521	0.119	8	0.578	0.181	8	0.970	0.178	8	0.732	0.255	8	a, b	^19^
16	1-Apr-2015	0.375	0.095	8	0.439	0.080	8	0.964	0.125	8	0.760	0.106	8	a, b	^19^
17	14-Feb-2016	0.332	0.089	8	0.557	0.068	8	0.920	0.165	8	0.759	0.123	8	a, b	u.p.
18	4-Apr-2016	0.277	0.122	8	0.383	0.130	7	0.968	0.141	8	0.720	0.159	8	a, b	^21^
19	23-May-2016	0.280	0.098	8	0.418	0.130	8	0.924	0.113	8	0.731	0.112	8	a, b	^21^
20	20-Jun-2016	0.434	0.096	8	0.504	0.118	8	0.998	0.163	8	0.773	0.089	8	a, b	^21^
21	28-Jul-2016	0.244	0.114	8	0.520	0.084	8	0.859	0.102	8	0.715	0.126	8	a, b	u.p.
22	24-Oct-2016	0.273	0.153	8	0.507	0.097	8	0.892	0.098	8	0.740	0.082	8	a, b	^22^
23	12-Jan-2017	0.274	0.090	8	0.520	0.118	8	0.911	0.082	8	0.742	0.117	8	a, b	u.p.
24	10-Mar-2017	0.244	0.117	8	0.480	0.169	8	0.905	0.095	8	0.748	0.123	8	a, b	u.p.
25	28-Apr-2017	0.417	0.144	8	0.510	0.165	8	0.911	0.100	8	0.726	0.138	8	a, b	u.p.
26	6-Aug-2017	0.222	0.115	8	0.389	0.112	8	0.950	0.064	8	0.791	0.090	8	a, b, c	u.p.
27	10-Nov-2017	0.383	0.122	8	0.472	0.082	8	1.137	0.156	8	0.870	0.103	8	a, b, c, d	u.p.
28	5-Apr-2018	0.332	0.172	8	0.353	0.069	8	0.890	0.225	8	0.741	0.123	8	a, b, c	u.p.
29	10-Aug-2018	0.401	0.102	8	0.491	0.091	8	0.913	0.143	8	0.736	0.072	8	a, b, c	u.p.
30	26-Dec-2018	0.285	0.101	8	0.403	0.102	8	0.964	0.100	8	0.852	0.082	8	a, b, c	u.p.
31	30-Oct-2019	0.324	0.107	8	0.435	0.049	8	0.850	0.048	8	0.753	0.046	8	a, b, c	u.p.
32	15-Dec-2019	0.314	0.105	8	0.379	0.087	8	0.822	0.083	8	0.721	0.073	8	a, b	u.p.

Information of 32 tests conducted from 2011 to 2019 were utilised for the pooled analysis. The 19 tests that included the four standard groups are from 2014 (Test Nos. 14-32).

*unpublished.

**Figure 2:**
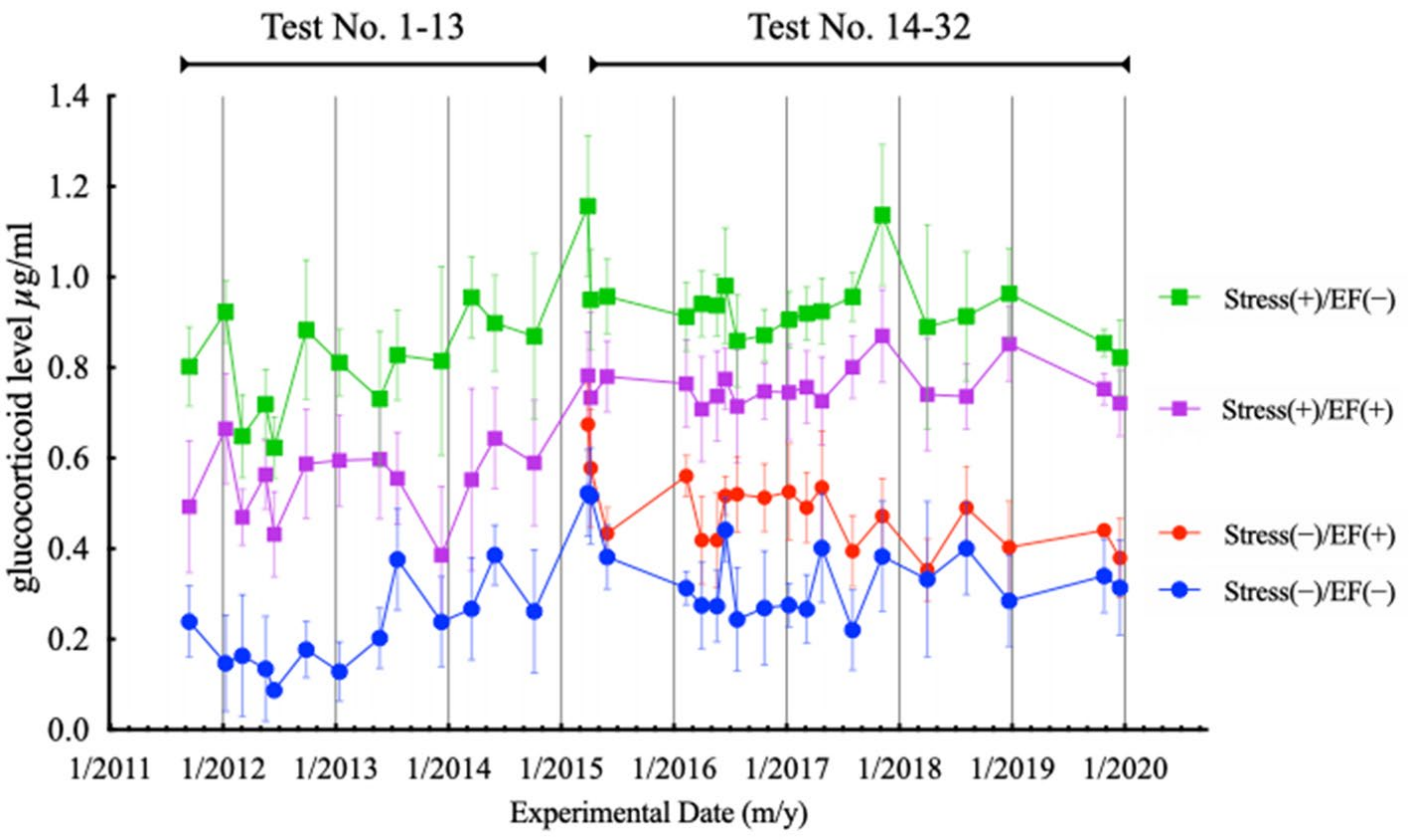
Effect of immobilisation and electric field exposure on immobilisation-induced increase in the plasma glucocorticoid level. Data of 32 tests conducted from 2011 to 2019 were pooled. The 32 tests included both published and unpublished data. The results are expressed as mean ± standard deviation.

We pooled and analysed the results of 32 tests conducted between 2011 and 2019 (n = 247, 149, 248, and 247, respectively, Table 1, note b). The results of Kolmogorov–Smirnov test indicated that the data were non-normally distributed. The results of Kruskal–Wallis test was significant (*P* < 0.0001; Fig. 2). The results of Dunn's multiple comparison test showed that there was no significant difference in the GC level between the stress(−)/EF(+) and stress(−)/EF(−) groups (Fig. 2). The GC level in the stress(+)/EF(+) group was also significantly higher than that in the stress(−)/EF(−) group (*P* < 0.0001), and its level in the stress(+)/EF(+) group was lower than that in the stress(+)/EF(−) group (*P* < 0.005; Fig. 2). The four common groups for the 32 tests were ranked as follows: stress(+)/EF(−) > stress(+)/EF(+) > stress(−)/EF(+) > stress(−)/EF(−).

### Haematology

**Figure 3:**
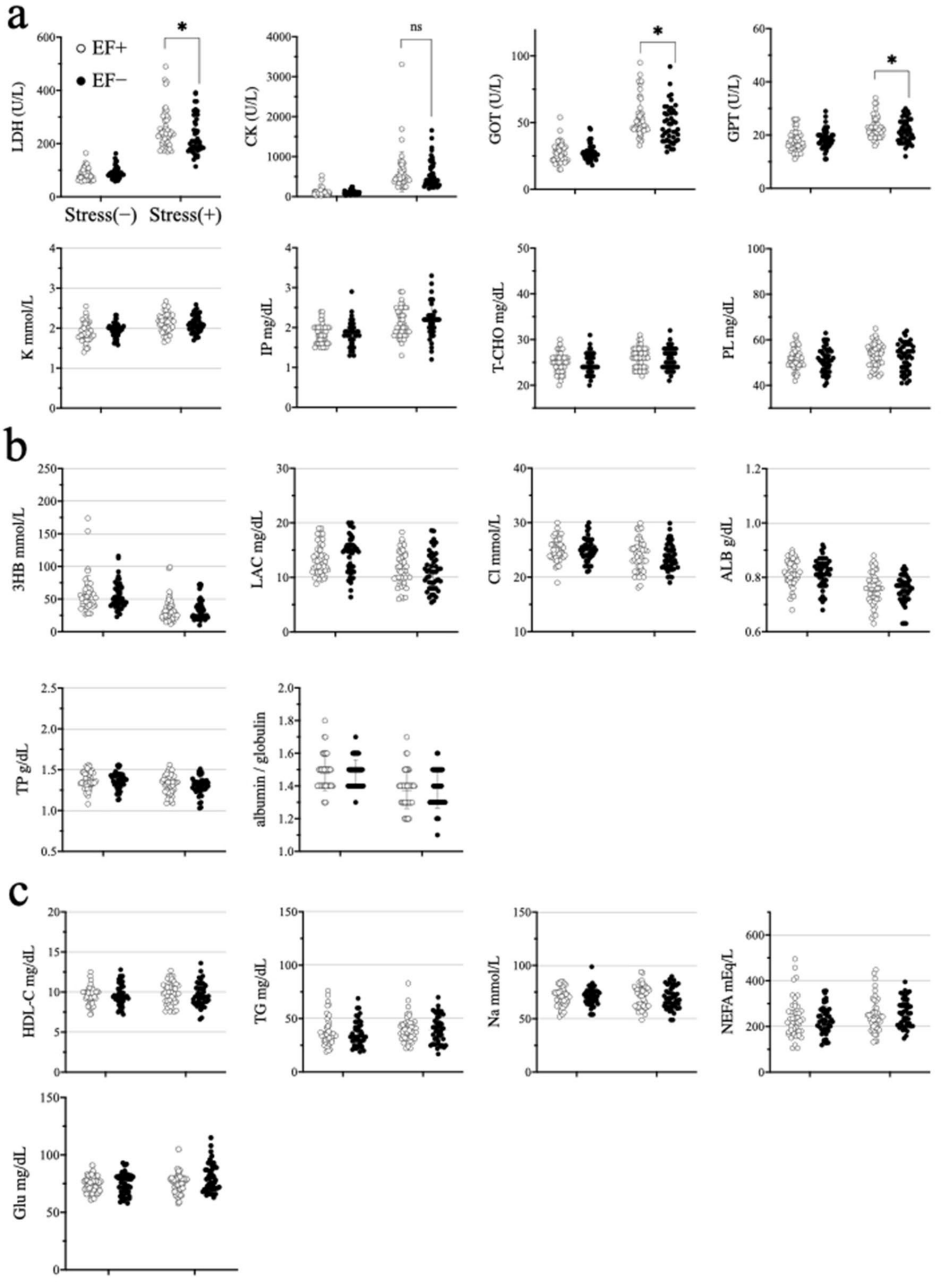
Effect of immobilisation and electric field exposure in biochemical tests (n = 46). (**a**) Up-regulated by immobilisation; (**b**) down-regulated by immobilisation; (**c**) not changed.

Using plasma samples from six tests (Test nos. 26–31 in Table 1, note c), 19 biochemical indexes were analysed using the normality test (Kolmogorov–Smirnov test), Kruskal–Wallis test, and Dunn's multiple comparison test, using the frozen plasma samples. The results of Kolmogorov–Smirnov test indicated that the data of 19 indexes were non-normally distributed. The EF alone (stress(−)/EF(+)) did not affect any index. Owing to stress, 8 indexes significantly increased (Fig. 3a), 6 indexes significantly decreased (Fig. 3b), and 5 indexes did not show any significant difference in the treatment groups compared with those in the non-treatment group (Fig. 3c). Among them, lactate dehydrogenase (LDH), glutamic oxaloacetic transaminase (GOT), and glutamic pyruvic transaminase (GPT) significantly differed between the immobilisation-alone (stress(+)/EF(−)) and co-treatment (stress(+)/EF(+)) groups (Fig. 3a), as determined using Dunn’s multiple comparison test.

### Metabolome analysis

**Figure 4:**
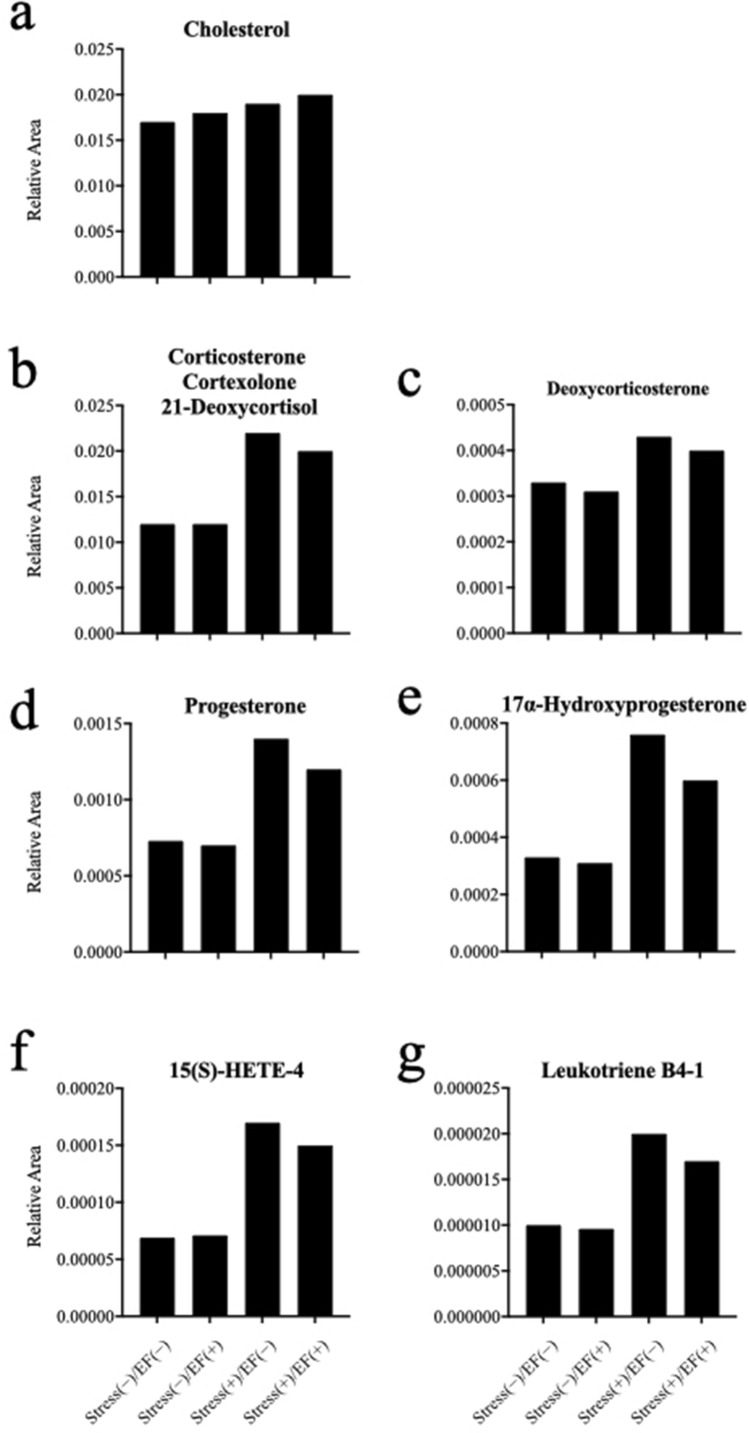
Metabolome analysis by liquid chromatography time-of-flight mass spectrometry. (**a**) Cholesterol; (**b**) corticosterone; (**c**) deoxycorticosterone; (**d**) progesterone; (**e**) hydroxyprogesterone; (**f**) 15(S)-HETE; (**g**) leukotriene B4. Plasma samples from the test (No. 27 in Table 1) were pooled for each treatment (n = 1) and analysed.

A metabolome analysis was performed using pooled plasma from one experiment (Table 1, note d). The changes in corticosterones showed a pattern similar to those of GC, but the cholesterol level did not differ among the four treatments (Fig. 4a–c). In addition, the changes in the plasma level of leukotrienes and hydroxyeicosatetraenoic acids (HETEs) (Fig. 4f,g), and progesterones (Fig. 4d,e) showed a pattern similar to those of GC.

## Discussion

In the present study, we elucidated the potential application of ELF-EF in medical field by performing a pooled analysis of previously published results obtained in an immobilisation-induced stress mouse model. Stress response was induced by the immobilisation of mice in a centrifuge tube for 30 min. The plasma GC level in the immobilised mice, that is, the stress(+)/EF(−) group, was approximately threefold higher than that in the control group, suggesting that immobilisation activated the endocrine system of the pituitary–adrenocortical axis and/or the sympathetic–adrenomedullary system^17,23,24^.

The plasma GC level increased in male BALB/c mice subjected to both immobilisation stress and exposure to 50 Hz EF, applied via the upper electrode of a parallel plate electrode system, and the GC level was lower than that in the immobilisation-alone group. The suppressive effect of EF on the induced stress response was dependent on both intensity (kV/m) and exposure time^18^. Furthermore, the effect was considerable at 20 min after the initiation of immobilisation^19^ and possibly depended on EF intensity distribution in the exposed regions of varied sizes on the body surface, even when the EF strength remained unchanged^21^. Similar effects were observed between EF of 50 and 60 Hz, which are the standard power frequencies in most regions, despite varied environmental brightness levels during EF exposure^21^, and sex and age differences in the mice^5^. As the suppressive effect of ELF-EF on immobilisation-induced increase in the GC level was clearly reproduced, these results suggest the robustness of this animal model to assess the efficacy of biological mechanisms induced by EF exposure (Fig. 1).

In contrast, the GC level in mice treated with ELF-EF alone was approximately 1.25-fold higher than that in the sham-controlled mice. This novel finding suggests that ELF-EF may act as a potential stressor. In a study by de Bruyn and de Jager^25^, the plasma level of corticosterone (41.9 ± 22.8 ng/mL) in 6-month-old male mice increased by approximately 3.26 times following exposure to an EF of 10 kV/m for 22 h^25^. Compared with the results of de Bruyn and de Jager, the magnitude of increase in the GC level in the current study (Fig. 1) was considerably small. It has been reported that electric shock from a short circuit between a charged mouse and the exposure system could provoke a stress response in the animal^26^, but this phenomenon was not observed in our study. EF may not act as a stressor but instead may function as an activating stimulus, such as plasma GC level increase after waking up in the morning in humans, which follows a circadian rhythm triggered by light stimulus. Further research is necessary to determine the role of EF-alone treatment in GC level increase.

The present study confirmed that 50 Hz EF of 10 kV/m can exert an anti-stress effect in mice. The results of this study suggest that the experimental approach adopted here, with respect to both technical and experimental design, may have a wider application in other studies on the biological effects of EF in different organisms. For example, a 0.1-mm thick polypropylene sheet placed between the lower electrode and the animal completely annihilated the suppressive effect of the EF on stress-induced GC level increase, and the effect reappeared as the sheet thickened^20^. Further development of the current method is necessary for extrapolation to humans.

Our experimental model had a few limitations. In all experiments, an EF intensity of 10 kV/m was selected based on a previous study that assessed EF intensities ranging from 2.5 to 200 kV/m, and 10 kV/m EF showed the highest anti‐stress effect^18^. Another reason for selecting this range was EF of intensity greater than 50 kV/m generated vibration and/or noise^18^. The effects of vibrations and/or noise were difficult to distinguish from those of the EF, thereby deterring the exclusion of a possible artefact at higher intensities. Therefore, this issue should be addressed and resolved to ensure that higher EF intensities can be further investigated.

The activities of LDH, GOT, and GPT differed between the immobilisation-alone (stress(+)/EF(−)) and co-treatment (stress(+)/EF(+)) groups (Fig. 3a). In each case, the increase caused by immobilisation was suppressed by the EF, similar to its effect on GC. There was a significant correlation among these three indexes in all combinations (Fig. S1a). Generally, a common feature of these three indexes is an increase in their blood levels during tissue or organ injury with inflammation. In addition, it is well known that GC reduces inflammation^27–31^. These results suggest the effect of ELF-EF on the association between stress and tissue damage involving inflammatory events, but there were no correlations between the three enzymes and GC level (Fig. S1b). Further investigation is necessary to confirm whether inflammation induced by GC can be alleviated by a reduction in the GC level due to ELF-EF exposure.

**Figure 5:**
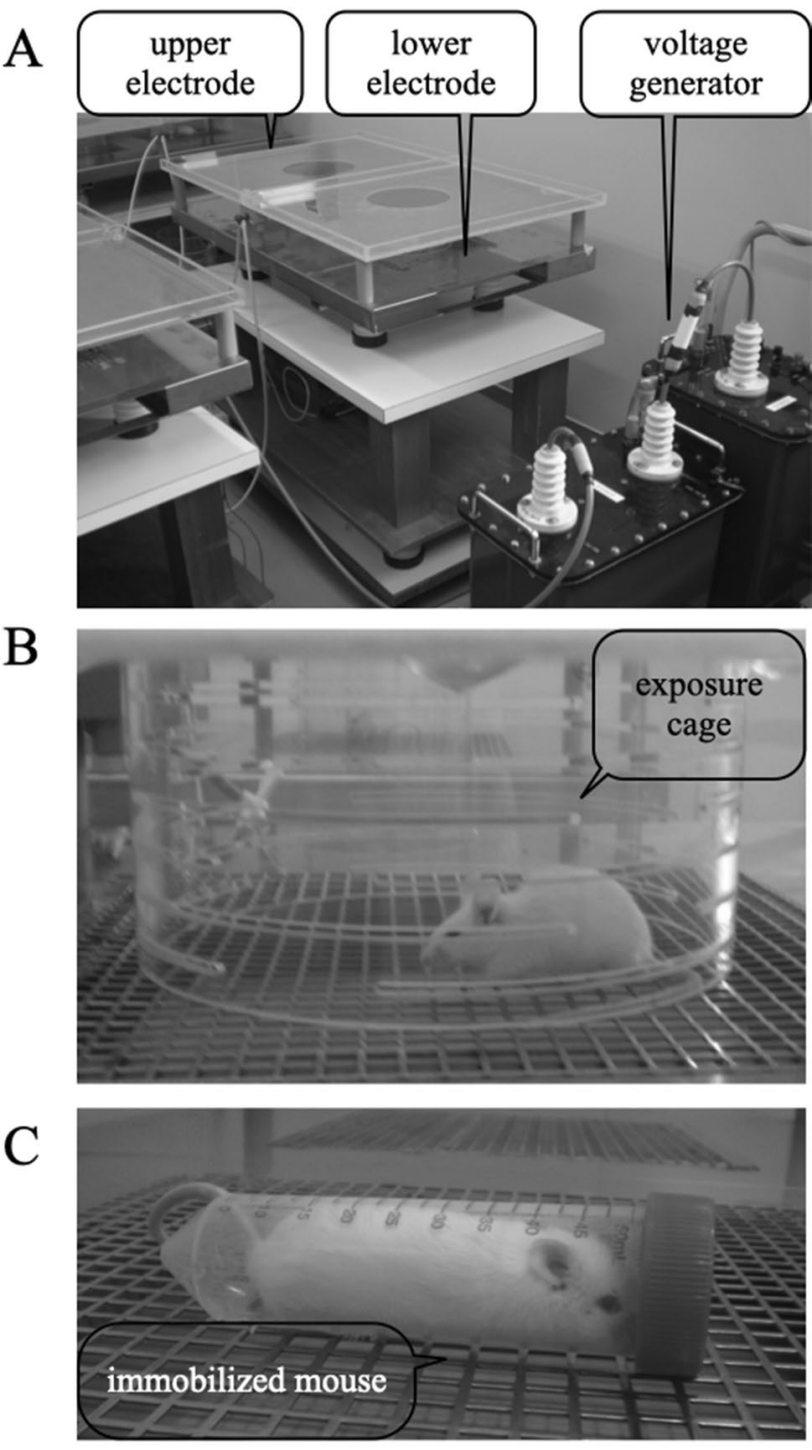
Electric field exposure system. (**a**) Voltage generator and parallel plate electrodes; (**b**) mouse in an exposure cage; (**c**) mouse in the 50-mL centrifuge tube restraining system.

The purpose of the metabolome analysis of the pooled plasma samples was to understand the GC-related pathways influenced by ELF-EF (Fig. 4). It was expected that the change trend of corticosterone might be similar to that of GC (Fig. 5b,c). The level of cholesterol, which is a source of corticosterone, did not change following immobilisation or EF exposure (Fig. 4a). In addition, ELF-EF did not affect the level of pregnenolone, a corticosterone precursor, although an increase was observed because of immobilisation (data not shown). These data suggest that ELF-EF does not inhibit the synthesis of corticosterone, but instead inhibits its release from the adrenal gland. A pattern similar to that of GC was observed for the female sex hormone progesterone (Fig. 4d,e). Progesterone is biosynthesised from cholesterol, and although it is important for the synthesis of corticosterone, the biological significance of the inhibition of progesterone synthesis must be evaluated in future studies. Furthermore, the levels of HETE and leukotrienes (Fig. 4f,g), which are indicators of inflammation^32,33^, increased owing to immobilisation. Their suppression by ELF-EF supports the possibility that the suppressive effect of ELF-EF on induced stress response may be involved in stress-induced inflammation. Because the metabolome analysis was conducted with one sample, analyses with more samples are necessary to confirm the trend. Nevertheless, further research is necessary to elucidate the hypothesis.

## Conclusions

Our results indicated that ELF-EF suppresses the plasma GC level under stress and slightly increases the GC level in a steady state condition. In addition, the plasma level of enzymes, which indicate tissue damage, was suppressed by ELF-EF. Therefore, the suppressive effect of ELF-EF exposure on the induced stress response appears to be involved in stress-induced inflammation in immobilised mice. These results demonstrate the treatment potential of ELF-EF.

The suppressive effect of ELF-EF on stress-induced elevation in the GC level showed high reproducibility (sensitivity (low error)). The animal model can be effectively used in future research on the biological effects of ELF-EF, even if the effects are not associated with stress response. We found that the blood level of markers of tissue damage or inflammation were elevated similar to endogenous steroids during immobilization, and that ELF-EF exerted an inhibitory effect on some of these markers. As stress causes several diseases, the results of this study will increase the use of ELF-EF for preventive or curative treatment. In addition, an increase in the blood level of GC by treatment with ELF-EF alone, although slightly, suggests that the biological effect of ELF-EF should be carefully interpreted from the perspective of not only biohazard but also medical application.

## Materials and methods

### Pooled data

Table 1 shows the date and a summary of information, including mean, standard deviation, and sample number for each pooled experiment conducted between 2011 and 2019, and the sample that was used for each analysis in this study, indicated as a–d.

### EF exposure system

The EF exposure system consisted of the following three major parts: a high-voltage transformer unit (maximum output voltage, 30 kV; Hakuju, Tokyo, Japan), a constant-voltage unit (CVFT1-200H; Tokyo Seiden, Tokyo, Japan) to avoid unexpected interferences from electrical noise originating from the commercial power supply, and an EF exposure cage^18–22^. The exposure system consisted of a cylindrical plastic cage (diameter, 200 mm; height, 100 mm) and two stainless steel electrodes (1,000 mm × 600 mm) that were placed above and under the cylindrical cage (Fig. 5a,b). The cylindrical cage had slits (length, 100 mm; width, 5 mm) throughout at intervals of 5 mm (Fig. 5b) to prevent smudges (from faeces or saliva) from disturbing the formation of a stable EF. To generate an EF of 10 kV/m in the cage, 50 Hz/1 kV was applied to the upper electrode, whereas the lower electrode was grounded. Separate cages and tubes were used for each animal; these were reused after being washed with a neutral detergent and completely dried. Using a digital thermometer placed on the lower electrode, the temperature was measured before EF exposure and at 10, 20, 30, 40, 50, and 60 min after EF exposure. The temperature inside the cage was 25 °C ± 3 °C during the period of EF or sham exposure. The temperature within the cylindrical cage did not change during either period. The humidity was maintained between 45 and 55%.

Mice in the EF‐treated groups were exposed to an EF of 10 kV/m for 60 min. To generate an EF of 10 kV/m in the cage, 50 Hz/1 kV was applied to the upper electrode, whereas the lower electrode was grounded. To measure field intensity and verify the system’s operation, an optical fibre voltmeter, which measures EF intensity using the Pockels effect, and an electro‐optic voltage sensor attached with a two‐cored Bi_12_SiO_20_ fibre (FOVM 03; Sumitomo Electric, Osaka, Japan) and a digital multimeter (Fluke 87; Fluke, Everett, WA) were used. The EF intensity was measured at 273 arbitrary points (21 × 13) on each cage floor. The 10 kV/m EF intensity applied to the cage had an error margin of ± 4% outside the exposure cage and ± 0.1% inside the cage.

A portable alternating current magnetic field metre (TMM‐1; Electric Power Engineering Systems, Kanagawa, Japan) was used to measure the magnetic field intensity in the area where each mouse was exposed to the EF. The magnetic field intensity was approximately 0.12 ± 0.04 mG when 50‐Hz, 10 kV/m EF was generated in the space.

### Animals

For all experiments shown in Table 1, 8-week-old male BALB/c mice were purchased from Charles River Japan (Kanagawa, Japan) and maintained in a pathogen-free environment at 24 °C ± 1 °C and 50% ± 10% humidity with daily artificial illumination (12-:12-h light/dark cycle with lights on from 07:00 to 19:00 h). The mice had free access to standard laboratory chow (CE-2; CLEA, Tokyo, Japan) and water, except during EF exposure and immobilisation.

**Figure 6:**
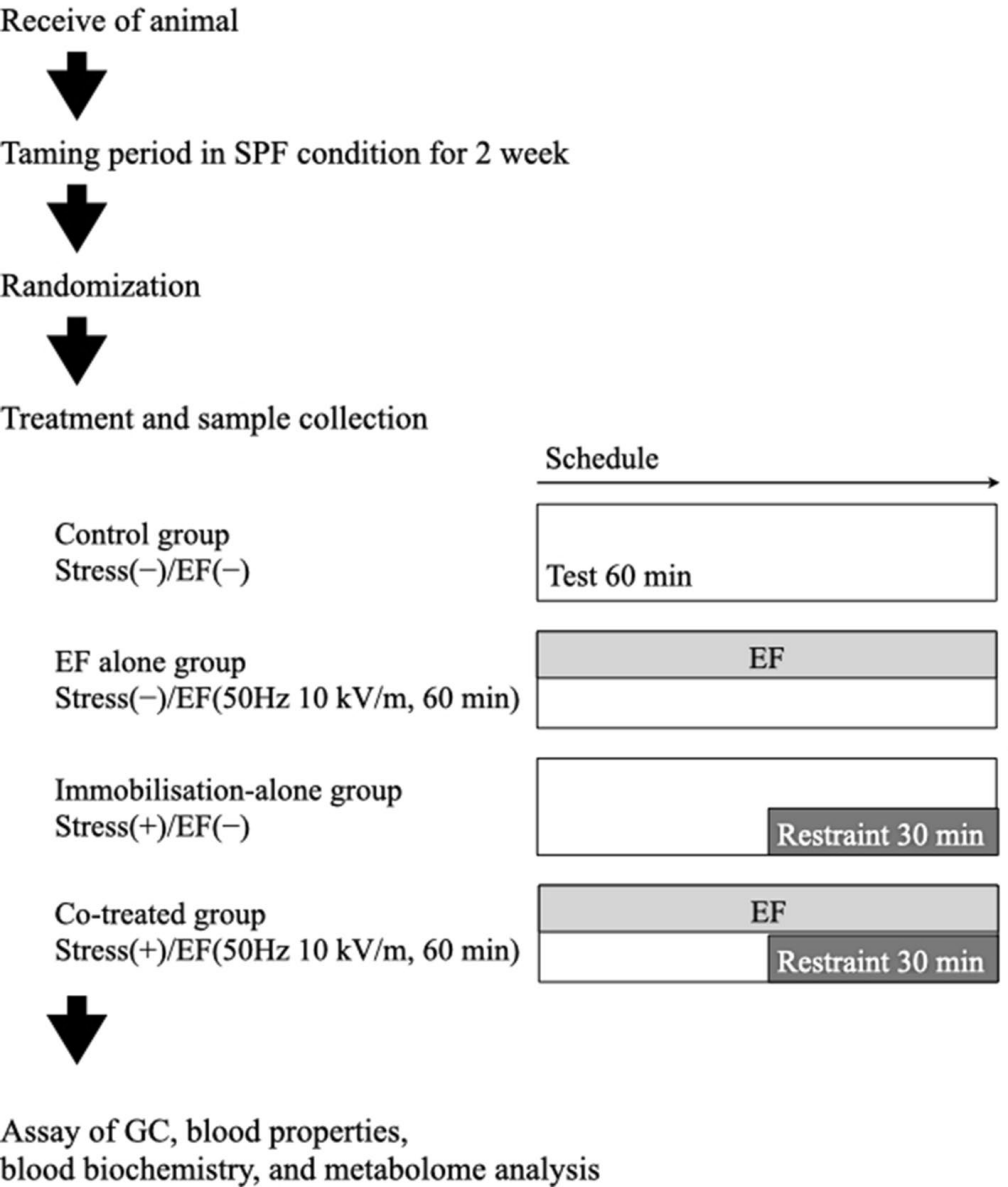
Experimental design employed to assess the influence of immobilisation and electric field (EF). After an acclimation period of 2 weeks, mice aged 10 weeks were divided into the following groups (n = 8 per group): control, stress(−)/EF(−); EF alone, stress(−)/EF(+); immobilisation alone, stress(+)/EF(−); and co-treatment stress(+)/EF(+). Mice in the co-treatment group were exposed to EF for 60 min and were immobilised during the second half (30 min) of the EF exposure period. In the control group, mice were handled in an identical manner, except that the EF condition was 0 V/m, and they were not immobilised.

To examine the effect of EF exposure, with or without immobilisation stress, on plasma GC level, male mice were divided into the following four groups (n = 8 each): control, stress(−)/EF(−); EF alone, stress(−)/EF(+); immobilisation alone, stress(+)/EF(−); and co-treatment, stress(+)/EF(+). Mice in the EF-treatment groups were exposed to the same EF (50 Hz, 10 kV/m for 60 min), and those in the co-treatment groups were immobilised during the second half (30 min) of the EF exposure period (Fig. 6). Mice in the control group were housed in the EF cage for 60 min without exposure to an EF (i.e., 0 V/m).

Stress was induced by the immobilisation of each mouse separately inside a 50-mL centrifuge tube (Nippon Genetics, Tokyo, Japan), which was placed on the lower electrode (Fig. 5c)^18–22^.

GC elevation as a stress response started immediately after animal immobilisation and peaked approximately 30 min later. When immobilisation was continued even after the peak in the GC level, the GC level showed a gradual decrease. Therefore, to use the changes in the GC level as an indicator of stress, we reasoned that the GC level should be measured 30 min after the start of the stress treatment.

All animal experiments were carried out in accordance with the Guiding Principles for the Care and Use of Research Animals of Obihiro University of Agriculture and Veterinary Medicine, Japan. The protocol was approved by the Committee on the Ethics of Animal Experiments of Obihiro University of Agriculture and Veterinary Medicine (Permit number 25-86, 25-155, 26-119, 27-132, 28-152, 29-164, 30-95, 19-162).

### Measurement of plasma GC and haematological analysis

Immediately after EF treatment (between 10:00 and 12:30 h), 800 µL of blood was collected from each mouse under 3% isoflurane anaesthesia (Mylan, Tokyo, Japan) and centrifuged at 1500×*g* for 10 min at 4 °C, and then the plasma was collected and stored at − 80 °C until use.

The stocked plasma from 19 tests was utilised for the pooled analysis of plasma GC (Test Nos. 14–32, Table 1). To derivatise GC for analysis, 200 μL of plasma was mixed with 900 µL of isooctane (2,2,4-trimethylpentane; Wako, Osaka, Japan) followed by vortexing and centrifugation at 380×*g* for 5 min at 24 °C. The upper layer was discarded, and then 900 µL of chloroform (Wako) was added to the lower layer. The sample was vortexed and centrifuged at 380×*g* for 5 min at 24 °C. The upper and white membranous layers were removed, and the lower layer was retained for analysis. Eight hundred microlitres of the lower layer was transferred to a new tube and mixed with 320 µL of solution containing 65% concentrated sulphuric acid and 35% ethanol (both from Wako), and then vortexed. The solution was incubated in dark for 3.5 h, and the fluorescence intensity of the sample was measured at 519 nm with excitation at 475 nm using a spectrofluorophotometer (RF-5300PC; Shimadzu, Kyoto, Japan). This method that utilises sulphuric acid-induced fluorescence of GC could be used to measure total GC^24^.

In addition, for the biochemical tests, 19 biological indexes (lactate dehydrogenase, creatine phosphokinase, GOT, GPT, chlorine, glucose, total cholesterol, phospholipid, potassium, inorganic phosphorus, sodium, polyhydroxybutyrate, lactate, high-density lipoprotein cholesterol, triglyceride, free fatty acid, total protein, albumin, and albumin-to-globulin ratio) were assayed using an auto-analyser (TBA-120FR; Toshiba, Tokyo, Japan). The stocked plasma samples from six tests were used for these assays (Test Nos. 26–31, Table 1).

### Metabolome analysis

As 500 µL of plasma was necessary for metabolome analysis, the plasma samples collected from eight mice (Test No. 27 in Table 1) were pooled for each group. Five hundred microlitres of the pooled plasma sample was added to 1500 µL of 1% formic acid/acetonitrile containing an internal standard solution (Solution ID: H3304-1002; Human Metabolome Technologies, Inc., Tsuruoka, Japan) at 0 °C to inactivate enzymes. The solution was thoroughly mixed and centrifuged at 2300×*g* for 5 min at 4 °C. The supernatant was filtered using Hybrid SPE phospholipid 55,261-U (Supelco, Bellefonte, PA) to remove phospholipids. The filtrate was desiccated and dissolved in 100 µL of isopropanol/Milli-Q for liquid chromatography mass spectrometry. Metabolome analysis was carried out at a facility of Human Metabolome Technologies Inc. (Tsuruoka, Japan). Liquid chromatography time-of-flight mass spectrometry analysis was carried out using an Agilent LC System (Agilent 1200 series RRLC system SL) equipped with an Agilent 6230 Time-of-Flight mass spectrometer (Agilent Technologies, Waldbronn, Germany). The systems were controlled using Agilent G2201AA ChemStation software version B.03.01 for CE (Agilent Technologies). The cationic and anionic compounds were analysed using an ODS column (2 mm × 50 mm, 2 μm) according to a previously described method^34^.

### Statistical analysis

Normality of data distribution was evaluated using the normality test. The differences among the groups were evaluated using the one-way analysis of variance (non-parametric method) and those between two groups were evaluated using Dunn’s multiple comparison test. The correlations and regressions were assessed using Pearson correlation analysis and slope regression analysis, respectively. Significance was defined at *P* < 0.05. All statistical analyses were conducted using Prism Version 8 (GraphPad Software, La Jolla, CA).

## Supplementary information

Supplementary Information 1.

## Data Availability

We declare that all data supporting the findings of this study are available within the article.
