# Supplementary figures and images for: Extremely low-frequency electric field suppresses not only induced stress response but also stress-related tissue damage in mice
Source: Sci Rep. 2020 Dec 7;10:20930. doi: 10.1038/s41598-020-76106-1 (PMC7721718; doi:10.1038/s41598-020-76106-1)

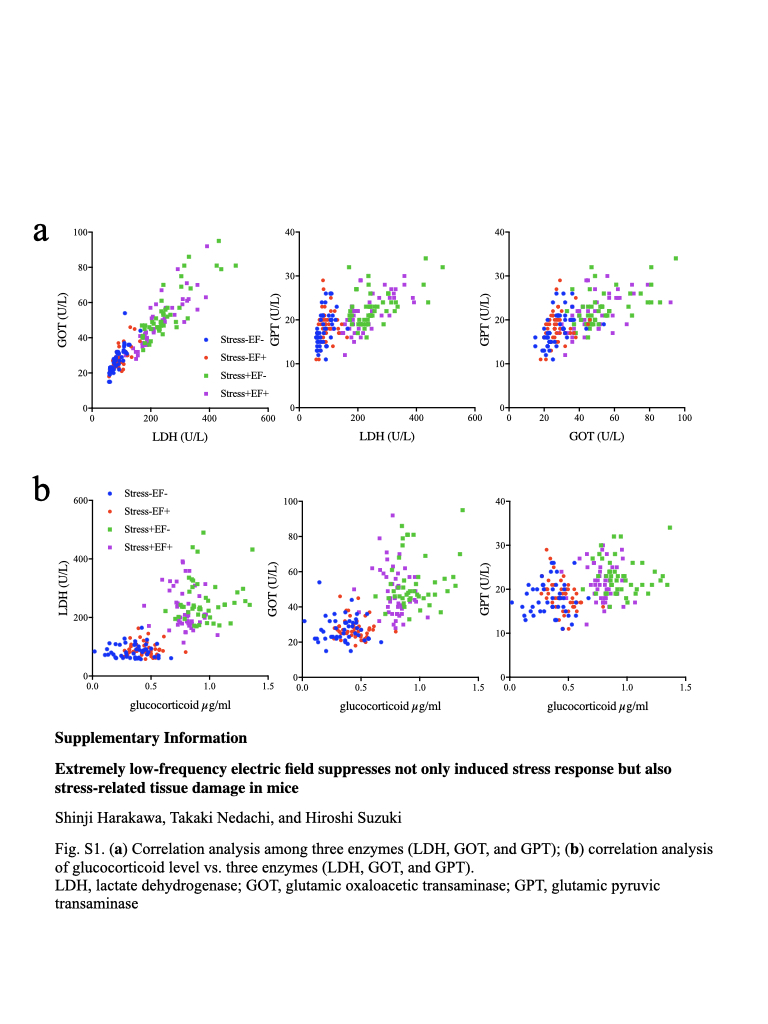

Supplement: Supplementary file 1 — Supplementary Information 1. [file 41598_2020_76106_MOESM1_ESM.jpg]
